# Bloodstream infection caused by *Erysipelothrix rhusiopathiae* serotype 6: Case report and literature review

**DOI:** 10.1016/j.idcr.2025.e02329

**Published:** 2025-07-24

**Authors:** Asumi Suzuki, Mariko Hakamata, Syunya Tanikawa, Naoto Kanno, Ikumi Yamagishi, Masahiro Ui, Hayato Tsuruma, Yuuki Bamba, Hideyuki Ogata, Satoshi Shibata, Hiromi Cho, Mizuho Sato, Nobumasa Aoki, Hiroshi Moro, Toshiaki Kikuchi

**Affiliations:** Department of Respiratory Medicine and Infectious Diseases, Niigata University Graduate School of Medical and Dental Sciences, Niigata, Japan

**Keywords:** *Erysipelothrix rhusiopathiae*, Bacteremia, Serotype, Spa typing

## Abstract

*Erysipelothrix rhusiopathiae* is a zoonotic, facultatively anaerobic, non-spore-forming, gram-positive bacillus. Although the serotypes and phylogenetic clades of *E. rhusiopathiae* strains isolated from animals have been shown to be closely related, the serotype identification of clinical isolates from human patients remains limited. We report a case of a 66-year-old woman with bacteremia caused by *E. rhusiopathiae* serotype 6. The patient had been admitted with aspiration pneumonia and heat stroke. She had no animal-related occupational history and had been receiving long-term oral prednisolone therapy for mixed connective tissue disease. Upon confirmation of *E. rhusiopathiae* positivity on blood cultures, she was treated with intravenous ampicillin for 10 days and achieved complete recovery. Agar gel precipitation and polymerase chain reaction tests identified the isolate as serotype 6. Surface protective antigen (Spa) typing by sequence analysis suggested a marine animal origin of infection. To the best of our knowledge, this is the first reported case of bacteremia caused by *E. rhusiopathiae* serotype 6 in humans. Spa typing through sequence analysis may provide variable information for identifying the infection source. *E. rhusiopathiae* carrying the *SpaB* gene should be considered in immunosuppressed patients, regardless of animal exposure history. Further studies are needed to elucidate the epidemiological distribution of serotypes among human clinical isolates.

## Introduction

*Erysipelothrix rhusiopathiae* is a facultatively anaerobic, non-spore-forming, gram-positive bacillus that causes zoonotic infections. The organism has been isolated from a wide range of wild and domestic animals, including fish, birds, and mice, with domestic swine serving as its primary reservoir [1]. Although it rarely causes human infections, *E. rhusiopathiae* is transmitted to humans through direct contact with infected animals or contaminated materials (including feces and urine), typically entering through skin abrasions or wounds [2]. This transmission route renders the infection more common among occupationally exposed individuals, such as fishermen, butchers, and cooks [3]. Human infections are categorized into three forms: localized cutaneous, generalized cutaneous, and septicemic [3], [4]. The localized cutaneous form is the most common, whereas the septicemic form is rare. *E. rhusiopathiae* is classified into serotypes 1a, 1b, 2, 4, 5, 6, 8, 9, 11, 12, 15, 16, 17, 19, 21, and N [5], with serotypes 1a, 1b, and 2 predominating in swine isolates [5]. However, the serotype identification of clinical isolates from human patients remains limited. The immunogenic surface protective antigen (Spa) protein of *E. rhusiopathiae* is a key virulence determinant that contributes to bacterial survival and host colonization. Although animal studies have demonstrated relationships between the Spa type, serotype, and phylogenetic clades, these associations remain undefined in human infections. To date, *E. rhusiopathiae* serotype 6 harboring the *spaB* gene has only been isolated from a wound in a patient with pyogenic spondylitis [6] but never from patients with septicemia. Herein, we describe the first case of a patient with bacteremia caused by *E. rhusiopathiae* serotype 6.

## Case report

A 66-year-old Japanese female was found immobilized at home in a high-temperature environment and transferred to the emergency department. Her medical history included mixed connective tissue disease (MCTD), interstitial pneumonia, pulmonary hypertension, aortic stenosis, and deep vein thrombosis. She had been diagnosed with MCTD, interstitial pneumonia, and pulmonary hypertension seven years prior to admission. Initial treatment consisted of prednisolone 50 mg daily, which was subsequently tapered to 5 mg daily, and she had been maintained on this dose along with azathioprine 50 mg daily until the current presentation. Although she kept a hamster as a pet, there was no recent history of hamster bites. She had no occupational animal exposure or recent history of handling raw fish or meat.

On admission, her vital signs showed a body temperature of 39.6 °C, blood pressure of 76/52 mmHg, heart rate of 117 beats/min, and oxygen saturation of 98 % while receiving supplemental oxygen at 5 L/min. Physical examination revealed Levine Ⅱ/Ⅵ systolic murmur at the right second intercostal space and bilateral fine crackles in the lower lung fields. Abdominal and neurologic examinations were unremarkable, and no skin lesions were observed. Laboratory tests showed elevated levels of leukocytes (9920/μL), aspartate aminotransferase (38 U/L), lactate dehydrogenase (344 U/L), creatinine (0.84 mg/dL), blood urea nitrogen (29 mg/dL), creatine kinase (615 U/L), and C-reactive protein (4.77 mg/dL). Chest computed tomography revealed infiltrative changes in the left lower lobe that were consistent with aspiration pneumonia. Sputum culture yielded *Escherichia coli* (1+), *Klebsiella pneumoniae* (1+), and methicillin-sensitive *Staphylococcus aureus* (1+). Antimicrobial susceptibility testing revealed that all isolated organisms were susceptible to conventional antibiotics.

**Fig. 1:**
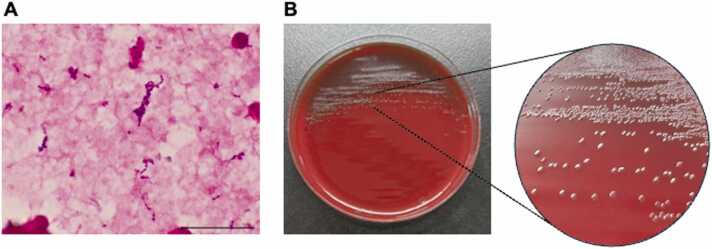
Gram stain image and colony morphology of *Erysipelothrix rhusiopathiae*. (A) Gram stain image of the positive blood culture obtained on day 4 of hospitalization showing gram-positive rods. Scale bar, 20 μm. (B) Small alpha-hemolytic colonies of *E. rhusiopathiae* on sheep blood agar after 48 h of incubation.

**Fig. 2:**
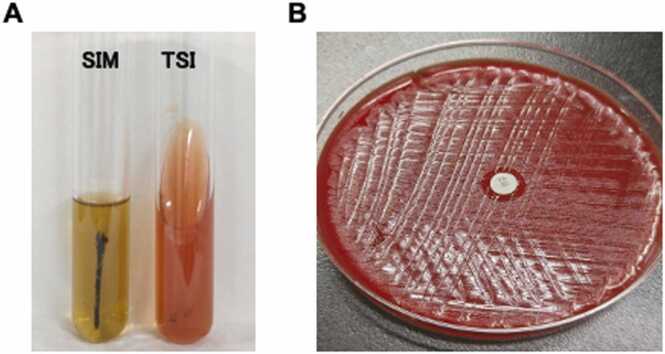
Biochemical and antimicrobial susceptibility characteristics of *Erysipelothrix rhusiopathiae*. (A) Hydrogen sulfide production indicated by black precipitate formation in sulfide indole motility (SIM) and triple sugar iron (TSI) media after 72 h of incubation. (B) Growth of the *E. rhusiopathiae* isolate adjacent to the vancomycin (30 μg) disk, indicating vancomycin resistance.

On the basis of the clinical presentation and laboratory findings, the patient was diagnosed with heat stroke and sepsis from aspiration pneumonia. Following the collection of two sets of blood cultures, intravenous ampicillin/sulbactam 3 g every 12 h was administered. On the second day after admission, she developed progressive hypotension and hypoxemia, requiring vasopressor support and noninvasive positive-pressure ventilation. Antimicrobial therapy was escalated to piperacillin/tazobactam 4.5 g every 8 h together with vancomycin. Gram-positive rods were isolated from two blood cultures on the fourth day after admission (Fig. 1A, B). The isolated colony was analyzed using matrix-assisted laser desorption/ionization (MALDI)-time-of-flight mass spectrometry (VITEK MS, Brochure) and identified as *E. rhusiopathiae* with 99.9 % probability according to the MALDI Biotyper version 3.0 database. Additional biochemical testing indicated that the isolate was oxidase and catalase negative, hydrogen sulfide positive on both sulfide indole motility and triple sugar iron media (Fig. 2A), and vancomycin resistant (Fig. 2B), characteristics consistent with *E. rhusiopathiae*. The serovar of the *E. rhusiopathiae* isolate was analyzed using conventional agar gel precipitation [7] and polymerase chain reaction tests [8] and identified as serotype 6. Sequence analysis of the DNA fragment [9] revealed that the isolate carried a *spaB* gene. Antimicrobial susceptibility testing was not available at our institution. Following the identification of *E. rhusiopathiae* on the sixth day after admission, the treatment was narrowed to ampicillin 2 g every 6 h. Serial transthoracic echocardiograms showed no evidence of infective endocarditis, and follow-up blood cultures remained sterile. After completing 16 days of antibiotic therapy, the patient recovered fully. She was transferred to a long-term care facility on day 57 of hospitalization but died 28 days later as a result of pneumatosis intestinalis.

## Discussion

*E. rhusiopathiae* infections typically manifest as a localized cutaneous infection known as erysipeloid. Systemic infections with bacteremia, with or without endocarditis, are uncommon. Some studies suggest that endocarditis complicates up to 90 % of bacteremia cases [10], [11], whereas others report a lower incidence of approximately 20 % [12]. A review of 22 cases of bacteremia without endocarditis revealed diverse patient populations, ranging from previously healthy individuals to those with solid tumors, hematologic malignancies, or steroid-treated systemic lupus erythematosus [12]. The mortality rate among patients with endocarditis was reported to be significantly high (33 %), whereas all patients with sepsis without endocarditic complications exhibited favorable outcomes with complete recovery [12]. Although 73 % of these patients reported prior animal or seafood exposure [12], infections can occur without animal exposure [13], [14]. Risk factors in non-occupational-related cases include immunocompromising conditions, such as diabetes mellitus, chronic kidney disease, and high-dose steroid therapy [12]. In the case of our patient, despite the absence of occupational animal exposure, her chronic state of immunosuppression due to the corticosteroids and immunosuppressive therapy for MCTD likely increased her susceptibility to infection.

Penicillin is the first-line antibiotic for *E. rhusiopathiae* infections, although effective alternatives include imipenem, cephalosporins, quinolones, clindamycin, and tetracycline [15]. Our clinical experience has confirmed the therapeutic efficacy of ampicillin. Notably, *E. rhusiopathiae* exhibits intrinsic resistance to vancomycin [15], a commonly used empiric therapy for gram-positive bacterial infections. These characteristics underscore the importance of accurate pathogen identification. However, the laboratory identification of *Erysipelothrix* species presents significant diagnostic challenges. The colonies are pinpointed and alpha-hemolytic (Fig. 1B), and their microscopic morphology as short gram-positive rods can be easily misidentified as streptococci or enterococci. The production of hydrogen sulfide, which was indicated in the isolate from our patient, serves as a distinct characteristic for distinguishing *E. rhusiopathiae* from other similar gram-positive bacilli, including *Corynebacterium* species, *Listeria monocytogenes*, and *Lactobacillus* species.

Through whole-genome analysis, *E. rhusiopathiae* has been classified into three clades (1, 2, and 3) and an intermediate group between clades 2 and 3 [16]. Marine mammal isolates predominantly belong to clade 1, whereas swine and poultry isolates typically cluster in clades 2, 3, or the intermediate group [16]. The cell membrane-bound Spa protein of *E. rhusiopathiae* is recognized as the major protective antigen of the species [17]. Spa proteins are classified as SpaA, SpaB, and SpaC, which correlate with specific serotypes: SpaA is found in serotypes 1a, 1b, 2, 5, 8, 9, 12, 15–17, and 23; SpaB in serotypes 4, 6, 11, 19, and 21 [6], [18]; and SpaC in the remaining serotypes [6], [19]. In animal isolates, the Spa type, serotype, and phylogenetic clade demonstrate strong correlations. Strains in clade 1 (marine mammals) typically harbor the *spaB* gene, whereas those in clades 2 and 3 (swine and poultry) possess the *spaA* gene [6], [16]. However, these associations have not been well characterized in clinical isolates from humans. The isolate from our patient was identified as serotype 6 and carried the *spaB* gene. Although *spaB*-carrying strains are not frequently detected in diseased animals in Japan [20], [21] and are considered less virulent than their *spaA*-carrying counterparts, the presence of such a strain in our patient suggests their potential to cause invasive human infections.

*E. rhusiopathiae* strains carrying the *spaB* gene are commonly found in marine animals, suggesting such an animal was the likely infection source in our patient. However, although the microorganism typically enters through skin lesions, oral transmission has been documented [22]. Therefore, given that our patient had no direct contact with marine animals, oral transmission appears to be the more likely route. Even though our patient showed no gastrointestinal symptoms during hospitalization, her subsequent development of pneumatosis intestinalis suggests that long-term steroid therapy may have impaired her intestinal mucosal barrier, potentially facilitating the bacteremia.

To the best of our knowledge following a literature review, serotypes have been characterized only for eight cases (including our patient) of *E. rhusiopathiae* infection (Table 1) [6], [23], [24], [25], [26], [27]. Of these, only the serotype information was reported for three patients [23], [24], whereas other clinical information was provided for the other five individuals. Most of the reported patients were older adults (mean age ± standard deviation: 66.0 ± 9.5 years), with 60 % (n = 3) males and 40 % (n = 2) females among cases where gender was identified. Two patients were identified as fishermen by occupation. Comorbidities predominantly included immunocompromised conditions such as rapidly progressive glomerulonephritis, diabetes mellitus, hepatitis C, and MCTD. Two patients were receiving immunosuppressive therapy with steroids. With regard to infectious manifestations, infective endocarditis was observed in two patients (40 %), bacteremia in two patients (40 %), and pyogenic spondylitis in one patient (20 %). Blood cultures were the primary source of bacterial isolation (80 %, n = 4), with abscess documented for one patient. The reported serotypes were diverse, including 1b, 2, 2b, 3, 6, and 9. Most of the isolates belonged to clade 2 or 3, containing the porcine-associated *SpaA* gene, whereas only two of the serotype 6 isolates were classified as clade 1, which is typically associated with fish hosts. With regard to transmission routes, fish-related exposure was suspected in four patients, suggesting a potential association between fish-related transmission and an immunocompromised status. Antimicrobial therapy primarily consisted of penicillin and carbapenem. One fatality was reported, whereas most of the patients showed favorable outcomes with appropriate antimicrobial treatment. Our case demonstrates that appropriate antimicrobial therapy can lead to the successful resolution of *E. rhusiopathiae* bacteremia, even in immunocompromised patients. However, because our patient later died as a result of pneumatosis intestinalis, a condition commonly observed in immunosuppressed individuals, her demise underscores the importance of addressing comorbid conditions and carefully managing immunosuppressive therapy during post-infectious care. The only other patient infected by a serotype 6 isolate carrying the *spaB* gene developed pyogenic spondylitis; our patient represents the first reported case of bacteremia related to this serotype. Both patients lacked occupational exposure or direct animal contact but had an immunocompromised status. This suggests that *E. rhusiopathiae* serotype 6 may preferentially affect immunocompromised individuals without requiring direct exposure. Notably, previous cases implicated fish rather than pigs as infection sources, highlighting the need for particular attention to exposure to marine products in immunosuppressed patients.

**Table 1:** Summary of reported cases of *E. rhusiopathiae* infection with identified serotypes.

**Case number**	**Study**	**Age**	**Sex**	**Occupation**	**Comorbid conditions**	**Immunosuppressive therapy**	**Infection**	**Source of bacterial culture**	**Serotype**	**Spa type**	**Possible exposure**	**Antibiotic therapy**	**Outcome**
1	Cross et al. [23]	NR	NR	NR	NR	NR	NR	NR	2b	NR	NR	NR	NR
2	Shiraiwa et al. [24]	NR	NR	NR	NR	NR	NR	NR	1b	NR	NR	NR	NR
3	Shiraiwa et al. [24]	NR	NR	NR	NR	NR	NR	NR	2	NR	NR	NR	NR
4	Kodera et al. [25]	67	M	Fisherman	NR	NR	Endocarditis	Blood	1b	NR	Fish	Ampicillin for 6 weeks	Cure
5	Yamamoto et al. [26]	58	M	Fisherman	Rapidly progressive glomerulonephritis	Methylprednisolone (1000 mg/day) for 3 days, followed by oral prednisolone (40 mg/day)	Endocarditis	Blood	3	NR	Fish	Meropenem for 2 weeks and panipenem/betamipron for 2 weeks	Death
6	Taguchi et al. [6]	80	F	NR	Type 2 diabetes, hepatitis C	NR	Pyogenic spondylitis	Abscess	6	SpaB	Fish	NR	Cure
7	Mileto et al. [27]	56	M	NR	Obesity, dyslipidemia, hypertension, hepatic steatosis	NR	Bacteremia	Blood	9	SpaA	Unknown	Piperacillin/tazobactam for 13 days	Cure
8	This study	66	F	None	Mixed connective tissue disease, pulmonary hypertension, aortic stenosis, deep vein thrombosis	Oral prednisolone (5 mg/day), azathioprine (50 mg/day)	Bacteremia	Blood	6	SpaB	Fish	Ampicillin for 10 days	Cure

**Note.** NR, not reported.

In conclusion, we present the first case of a patient with bacteremia caused by *E. rhusiopathiae* serotype 6. Our findings indicate that *E. rhusiopathiae* strains harboring the *spaB* gene can cause invasive human infections, particularly in immunosuppressed hosts. Further research is warranted to elucidate the relationship between *E. rhusiopathiae* serotypes and clinical manifestations in human infections.

## CRediT authorship contribution statement

**Satoshi Shibata:** Writing – review & editing. **Hideyuki Ogata:** Writing – review & editing. **Mizuho Sato:** Writing – review & editing. **Asumi Suzuki:** Writing – original draft, Data curation. **Hiromi Cho:** Writing – review & editing. **Hayato Tsuruma:** Writing – review & editing. **Masahiro Ui:** Writing – review & editing. **Yuuki Bamba:** Writing – review & editing. **Hiroshi Moro:** Writing – review & editing, Supervision. **Syunya Tanikawa:** Supervision, Investigation. **Nobumasa Aoki:** Writing – review & editing, Supervision. **Mariko Hakamata:** Writing – review & editing, Writing – original draft, Data curation, Conceptualization. **Ikumi Yamagishi:** Writing – review & editing. **Toshiaki Kikuchi:** Writing – review & editing, Supervision. **Naoto Kanno:** Writing – review & editing.

## Consent

Written informed consent was obtained from the family of the patient for publication of this case report and any accompanying images. A copy of the written consent is available for review by the Editor-in-Chief of this journal on request

## Ethical approval

Written informed consent was obtained from the family of the patient for publication of this case report and any accompanying images. Ethical approval was not required for this work.

## Funding

This study was supported by a KAKENHI Grant-in-Aid for Young Scientists [23K15363] from the 10.13039/501100001691Japan Society for the Promotion of Science (to MH).

## Declaration of competing interest

The authors declare that they have no known competing financial interests or personal relationships that could have appeared to influence the work reported in this paper.
